# Assessment of tree‐associated atypical myopathy risk factors in *Acer pseudoplatanus* (sycamore) seeds and leaves

**DOI:** 10.1111/evj.14475

**Published:** 2025-01-25

**Authors:** Sonia González‐Medina, Carolyn Hyde, Yu‐Mei Chang, Richard J. Piercy

**Affiliations:** ^1^ Comparative Neuromuscular Diseases Laboratory, Department of Clinical Science and Services The Royal Veterinary College London UK; ^2^ Bio‐Analysis Centre London UK; ^3^ Comparative Biomedical Sciences The Royal Veterinary College London UK; ^4^ Present address: School of Veterinary Medicine and Science Nottingham University Nottingham UK

**Keywords:** *Acer*, horse, hypoglycin A, myopathy, sycamore

## Abstract

**Background:**

Sycamore tree‐derived hypoglycin A (HGA) toxin causes atypical myopathy (AM), an acute, equine pasture‐associated rhabdomyolysis but incidence fluctuates.

**Objectives:**

Investigate whether tree or environmental factors influence HGA concentration in sycamore material and are associated with AM relative risk.

**Study design:**

Retrospective and experimental prospective study.

**Methods:**

UK sycamore population, seed production and AM incidence data were obtained. HGA concentration was measured in seeds from trees from 10 different central UK locations. The effect of tar spot infection, seed maturity, tree trunk girth, location (urban/countryside), AM cases within 130 m, soil type, facing direction of seeds on the tree and year on seed HGA concentration was examined. HGA concentration was compared in whole and homogenated seeds stored in different ways.

**Results:**

HGA concentration in sycamore seeds was not associated with tree tar spot infection, location, trunk girth, seed weight or branch‐facing direction but HGA concentration in sycamore seeds varied significantly and in parallel year on year in the same trees. Trees in the same vicinity tended to have similar HGA concentrations in their seeds when compared with those from farther afield. Seed production estimates were positively correlated with regional AM case incidence (*τb* = 0.3; *p* = 0.007). HGA sycamore seed concentration remained stable as seeds matured, but HGA declined in leaves as they wilted in autumn. Warmer and wet storage resulted in higher HGA concentrations in seed homogenates but not in whole seeds. HGA was detected in water containing sycamore seeds for 48 h.

**Main limitations:**

Lack of accurate weather data; sampling restricted to central England.

**Conclusions:**

Tree factors that were investigated did not affect HGA concentration in sycamore seeds but HGA concentrations varied year on year. AM incidence is related to seed production; conditions that mimic browsing and ingestion increased seed HGA concentration. HGA toxicity could occur from contaminated water sources.

## 1 INTRODUCTION

Equine atypical myopathy (AM) is a toxic rhabdomyolysis caused by the disruption of muscle mitochondrial metabolism following ingestion of hypoglycin A (HGA) contained in plant material derived from some *Acer* tree species.^1^, ^2^, ^3^ The first description of equine atypical myopathy (AM) was in 1939,^4^ and reported case numbers have fluctuated over the years although there has been a steady increase in the number of cases reported from 2007^5^, ^6^ likely due in part to improved disease recognition.^7^ However, annual variation in AM incidence remains a striking disease feature: consequently, tree‐specific or regional factors might play a role. Most studies have focused on demonstrating the presence of sycamore trees in affected pastures and determining the concentration of HGA in *Acer pseudoplatanus* seeds, but other environmental factors might play an important role in the disease's epidemiology.

The typical biannual presentation of AM reveals the interaction between both horse and tree. Sycamore seeds and seedlings are accessible on the ground in autumn and spring respectively, when most horses spend the majority of their time grazing. However, sycamore seed production varies each year^8^, ^9^, ^10^, ^11^ and is dependent on different factors including tree age (sycamores begin producing seeds when aged around 25 years)^8^, ^12^; further, certain weather conditions can favour flowering, pollination and seed dispersal.^8^, ^9^ Additional factors affect the chemical composition and nutrient concentration of seeds and fruits, and the maturation process heavily impacts their biochemical constituents.^13^, ^14^ For example, HGA concentration in Ackee trees (*Blighia sapida*) decreases in the fruit aril as the fruit ripens but the opposite occurs in the fruit seed.^15^ Consequently, if the same is true in sycamore seeds, the presence of unripe seeds on the ground following strong winds^16^ might pose an increased AM risk.

Pollutants contained in soil, water and air as well as phyto‐infections trigger stress responses in plants^17^, ^18^, ^19^ which can alter growth and photosynthesis rates, as well as the production of substances that are deterrents for predators and pathogens.^20^, ^21^, ^22^, ^23^ *Rhytisma acerinum* (European tar spot fungus) commonly infects Acer trees in late summer causing circular black spots with yellow edges on the leaves. Although it is considered a mild biotrophic infection in European populations of *A. pseudoplatanus* and *A. platanoides*, it can reduce tree growth.^24^ In early AM epidemiological studies, this fungal infection was considered the possible primary cause of AM.^25^ Whilst more recent data revealed that it is the tree itself (rather than the fungus) that is toxic,^2^, ^26^ it remains conceivable that tar spot infection might influence HGA concentration in plant tissues.

In this study, we evaluated sycamore tree population expansion in the United Kingdom, and the effects of annual seed production and annual HGA seed variability on AM case reporting. Further, we examined environmental, age‐associated and stress‐associated factors that might impact *A. pseudoplatanus* seed HGA content. The aim of this work was to explain annual and seasonal AM variability and determine particular conditions that might exacerbate toxicity or increase AM risk.

## 2 MATERIALS AND METHODS

### 2.1 Distribution of *A. pseudoplatanus* in the United Kingdom

Occurrence data from two different tree species, *A. pseudoplatanus* (Sycamore) and *Fagus sylvatica* (European Beech) were retrieved from the National Biodiversity Network (NBN) website: https://species.nbnatlas.org. This website collects and assimilates data from different local sources concerning the presence of tree species: tree occurrence is collated into interactive maps with public access through the NBN atlas. Maps displayed in this article were elaborated with the interactive tool: selection of the range of years to display and an ordnance survey grid size of 10 km. The range of years for the maps was chosen to represent dates from the earliest accounts of AM to the present. Since the beech tree is considered native and is also widely distributed in the United Kingdom: it was used to control for possible reporting bias.

### 2.2 Association between *A. pseudoplatanus* fruit score and AM cases

The fruit score (a measure of seed production) from 2011 to 2015 was supplied by the Woodland Trust through the Nature's Calendar program for which information is collated from different observers in several UK counties. Fruit score is recorded by observers as follows: 1 = no fruit; 2 = meagre; 3 = moderate; 4 = good crop and 5 = exceptional. The average fruit score and maximum fruit score of all observations in that county were calculated. The number of AM cases reported by UK county and year were obtained from those reported to the Atypical Myopathy Alert Group website (http://www.myopathieatypique.be) in the same period. Kendall's tau‐b correlation was used to assess the correlation between the number of AM cases reported per county and the average fruit score observed. A Chi‐square test was used to assess differences in the number of cases reported in each fruit score category.

#### 2.2.1 Experimental work

HGA was measured in *A. pseudoplatanus* plant material selected to test various hypotheses regarding sources of toxin variation. Specifically, we examined the influence of seed weight and maturity, the facing direction of seeds on a tree (i.e. north, south, east or west); tree age and location. Furthermore, we compared HGA content in *Acer pseudoplatanus* plant material at different times of the year and over three successive years from the same trees. Details of each experiment are summarised in Table 1 and further explained in Methods S1 including the number of trees and seeds/tree per analysis and statistical methods. Most experiments analysed samples from individual trees, apart from those that followed a group of trees over time.

**TABLE 1:** Summary of experimental work.

	Seed weight	Seed maturity	Facing direction of seed within tree	Tree age/location/soil/Tar spot	HGA variation per season	HGA annual variation within tree	Moisture effect in sycamore seeds and homogenates	Water contamination with seeds
*N* (trees)	4	4	4	32	11	14 followed for 3 consecutive years	6	3
*n* (seeds/tree)	20	20/maturity condition Partially mature, mature and flat	20 (5 seed/bunch)	150 g seeds/tree Analysed = 1 g/seeds	150 g seeds/tree/leaves Analysed = 1 g/seeds/leaves	150 g seeds/tree Analysed = 1 g/seeds	150 g seeds/tree Analysed = 1 g/seeds/tree/Temp Homogenates × 6 trees Full seeds × 3 trees	High (4 seeds) and Low (2 seeds) concentration of seeds in 10 mL water. Seeds were either fresh or frozen
Statistics	Linear mixed effect model			General linear model	Paired *T*‐test	Repeated measure ANOVA	Mixed effects model followed by Fisher's least significant difference (LSD) pairwise comparison	Descriptive statistics

We also examined the effects of storage temperature (from −20°C to +20°C) and moisture exposure on HGA concentration in whole seeds and in seed homogenates in vitro. Finally, HGA concentration was measured in water that had been exposed to sycamore seeds (either fresh or frozen) for 48 h.

In all cases, HGA content in plant material was measured according to previously published methods.^27^

### 2.3 Data analysis

The relationship between HGA (or natural log of HGA if data were not normally distributed) and the following factors were examined: tar spot infection, seed weight and characteristics, tree trunk girth (surrogate for age), location (urban/countryside), reported cases of AM within 100 m, soil type, facing the direction of seeds on the tree, the distance between trees and year. Tree age was estimated from the trunk girth (girth/2.5 growth factor).^28^ Briefly, mixed effects models were used to account for repeated measures from the same tree if observations at the seed level were used for the data analysis; paired *t*‐test or repeated measures ANOVA were used to compare mean HGA concentrations between seasons or between years based on repeated observations at tree level. Linear models were used to evaluate the influence of tree age, location, soil types or tar spot on HGA levels. Histograms and analysis of residuals were used to assess normality. Data were logarithmically transformed when necessary to achieve normality before statistical analysis. Descriptive statistics were also performed when deemed appropriate for data summaries; mean ± SD, median (minimum–maximum) and coefficient of variation between individual trees (%CV). Statistical analyses were performed with SPSS 21 or GraphPad Prism 7.

A sample size of at least 10 trees was required to detect a mean difference of 445 ± 400 mg/kg HGA between spring and autumn with a 5% type I error rate and 80% power using data provided in Westermann et al.^29^ Since there was no reliable information available for the effects of seed weight, maturity, moisture on the HGA, a sample size of 4–6 trees were used based on the resource equation method.^29^, ^30^ Similarly, a sample size of 16–24 trees was required to evaluate the impact of environmental factors on HGA based on the resource equation method. Optimal *sample size* and adequate *power* to detect statistical significance in the seed position within the tree was estimated from data presented by Valberg et al.,^2^ using an HGA seed variation between 3 and 160 μg/g. Based on a power of 80% and alpha of 0.05, four bunches of seeds from three trees would enable the detection of a mean difference of 160 μg/g.

## 3 RESULTS

### 3.1 Distribution of *A. pseudoplatanus* in the United Kingdom and the association between *A. pseudoplatanus* fruit score and AM cases

**FIGURE 1:**
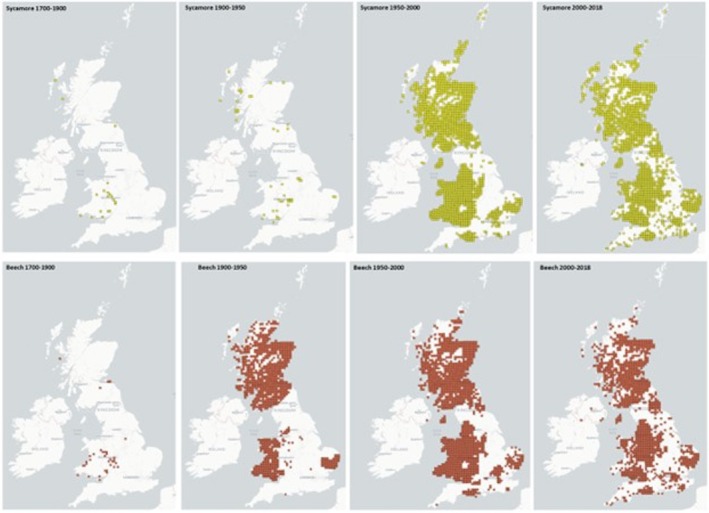
Tree recordings of two species over four different time intervals. The intervals were selected according to reports of AM in the literature. Data was sourced through the National Biodiversity Network (NBN) website and included several resources. Updated 15 January 2016. http://data.nbn.org.uk/ Accessed 6 October 2018. Data provided by this server refers to the presence of the tree/number of times that has been recorded and communicated to the NBN. Note that the first cases of AM occurred around 1939 in Wales where sycamore trees were recorded. Sycamore trees have spread over the United Kingdom from 1950 onwards when compared with beech trees, which have remained constant.

Both sycamore and beech trees were rarely recorded in the United Kingdom between 1700 and 1900. Thereafter, whereas the recording of beech trees remained relatively constant, there has been a dramatic increase in the number of sycamore trees in the United Kingdom from 1950 with the species now covering wide areas of Scotland, Wales, western counties of England, Dorset and East Anglia (Figure 1).

Fruit score data were available for 48 counties in England. Exactly 5% of AM cases (*n* = 11/222) were reported in counties with an average score of 3, 46% cases (*n* = 102/222) in counties with an average score of 4 and 27% (*n* = 61/222) in counties with an average score of 5. Fruit score data were not available for 25 counties in which 22% AM cases (*n* = 49/222) were reported. There was a moderate, positive correlation between the number of cases reported and the fruit score, which was statistically significant (*τb* = 0.3; *p* = 0.007). Significantly more AM cases were reported in counties with a fruit score of 4 or 5 (*p* = 0.02) than in other counties.

#### 3.1.1 HGA concentration variation within and between trees; seed weight, maturity, position within the tree and annual variation

Seed weight varied significantly (*p* < 0.0001) between partially mature seeds (0.3 g; 0.1–0.57 g; median and range) and the other groups: mature (0.21 g; 0.09–0.4 g) and flat seeds (0.07 g; 0.01–0.17 g). HGA was not found in sycamore seed wings (<LOD = below limit of detection). Flat seeds (0.54 μg; 0.05–9.89; *p* < 0.001) contained significantly less HGA than young (20.28 μg; 9.13–237.45 μg) or mature seeds (27.03 μg; 11.56–172 μg).

The concentration of HGA in seed bunches within the same tree was not associated with facing direction (i.e., north, south, east or west) (*p* = 0.8), seed maturity (*p* = 0.3) or seed weight (*p* = 0.9). HGA variation between bunches within the same tree was 5.5% while variation between seeds within the same bunch was 63.6%. This indicates that sampling of a single, full bunch is representative of the tree, but using a single seed might be misleading.

**FIGURE 2:**
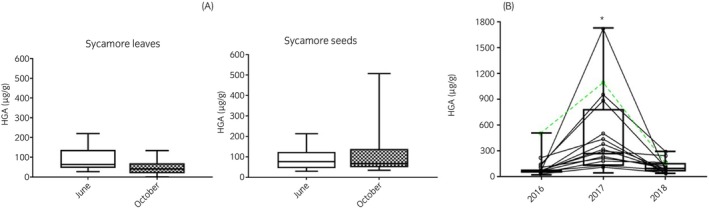
(A) HGA content in seeds and leaves from the same 11 trees in late spring (June) and autumn (October). Data are presented in whisker plots depicting HGA concentration at different time points. (B) HGA content of sycamore seeds (mid‐October) in the same 14 trees followed over 3 years. Connecting lines show the mean yearly HGA concentration in seeds from each individual tree. The tree represented in green was on the premises where an AM case was reported in 2016. Superimposed whisker plots show the distribution of total HGA concentration in different years. Significant differences (*p* < 0.05) are represented with asterisks.

HGA concentration was significantly lower in sycamore leaves collected from the same trees in October when compared with those from June (*p* = 0.002); however, it remained stable in seeds (*p* = 0.9) (Figure 2A). The median autumnal HGA concentration in seeds collected from the same trees was significantly higher in 2017 (*n* = 14; 305.5 μg/g; 428–1729 μg/g; median and min‐max) than in both 2016 (*n* = 14; 67.9 μg/g; 44.3–506.8 μg/g; *p* = 0.001) and 2018 (*n* = 14; 93.9 μg/g; 37.4–292.7 μg/g; *p* = 0.01) (Figure 2B).

### 3.2 Influence of local environment on seed HGA concentration

**FIGURE 3:**
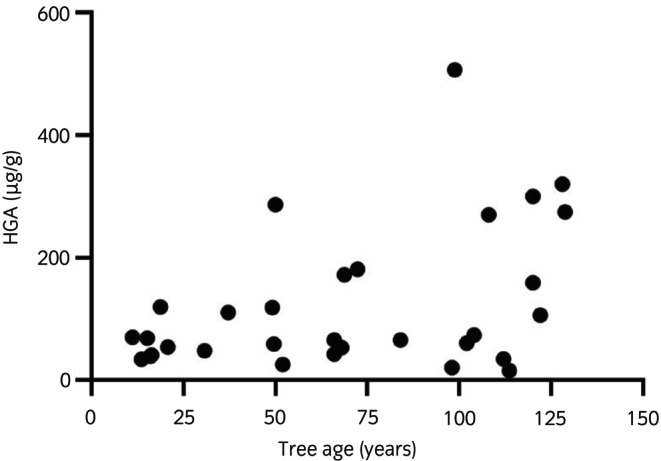
Association between estimated tree age and seed HGA content in the study population (32 trees). *r* = 0.32; *p* = 0.07.

Sixty‐five percent of trees evaluated were in urban areas (*n* = 21/32) and 78% were visibly infected with European tar spot (*n* = 25/32). Forty percent (13/32) of samples were from trees near AM‐associated pastures. The median trunk girth of trees was 167.5 cm (range: 28–322) and therefore the median estimated tree age of the study population was 67 years (11–129) (Figure 3). Slightly acid‐loamy soils were present in 25% (*n* = 8/32) of sampling areas, lime‐rich soils were found in 50% (*n* = 16/32), loamy and clay‐rich soils in 19% (*n* = 6/32) and base‐rich soils in 6% of sampling areas (*n* = 2/32). HGA concentration in sycamore seeds was not associated with any of the environmental factors investigated, namely fungal infection, urban versus rural location, tree estimated age, prior AM cases identified in the surrounding area or soil type (Table 2). Despite this, trees located within 10 m from each other had a significantly lower coefficient of variation (*p* = 0.002) between their individual seed HGA concentration (CV = 25% ± 0.08; *n* = 5) than those that were separated 1 km or more (CV = 68% ± 0.18; *n* = 4).

**TABLE 2:** Tree factors associated with HGA concentration in sycamore seeds from 32 trees.

	Fungal infection; *p* = 0.55		Tree location; *p* = 0.56		AM cases vicinity; *p* = 0.44		Soil type; *p* = 0.21			
	Yes	No	Urban	Countryside	Yes	No	Acid loamy	Lime rich	Loamy‐clayey	Base rich
HGA (μg/g)	64.5 (15.8–506.8)	65.6 (42.7–119.5)	64.5 (20.8–286.6)	68.6 (15.8–506.8)	59.5 (15.8–59.7)	65.6 (15.8–59.5)	119 (42.7–286.6)	53.6 (15.8–221.5)	56.8 (25.7–506.8)	92.1 (73.6–110.7)

*Note*: HGA concentrations are shown for each group as median (min–max). HGA was log transformed before analysis by general linear model. No significant associations were found between studied factors and HGA concentration in sycamore seeds.

### 3.3 Effect of temperature and moisture on HGA concentration of sycamore seeds

**FIGURE 4:**
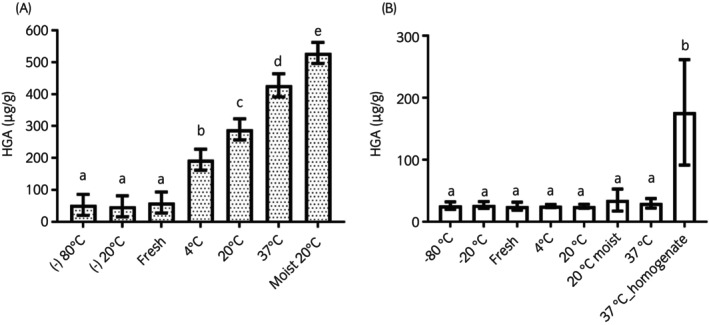
Effect of different storage conditions for 48 h on HGA content in seed homogenates (A) and whole seeds (B). Higher temperatures and moisture influenced the HGA content of seed homogenates but not in whole seeds (mean and SD; a, b/c/d/e: *p* < 0.001; b, c: *p* = 0.005; b, d/e: *p* < 0.001; c, d: *p* = 0.005; c–e: *p* < 0.001. *N* = 6 homogenates each from seeds derived from different trees). No significant differences were found between temperature conditions except with the control (those homogenised and exposed to 37°C for 48 h) (a, b: *p* < 0.001). Results are presented as mean ± SD of homogenised seeds derived from six trees.

Storage conditions had a marked effect on HGA content in homogenised seed material (Figure 4). Storage above freezing resulted in higher concentrations of HGA in seed homogenates when compared with control samples (*p* < 0.0001), with an increase in HGA with increasing temperature; moisture significantly raised the HGA content of seeds stored at 20°C. No differences were detected between freshly analysed homogenised samples and those stored at −20°C and −80°C. In contrast, storage temperature (above freezing) or when moist, did not influence HGA concentration in whole sycamore seeds stored for 48 h (*p* < 0.001).

#### 3.3.1 Water trough contamination

Only water in contact with seeds that had been frozen and then thawed contained HGA. For the latter, on average, HGA concentration in water was 1.27 ± 0.72 μg/mL in the largest number (4 seeds/10 mL water) and 0.2 ± 0.29 μg/mL at the lowest (2 seeds/10 mL water).

## 4 DISCUSSION

The production of seeds is an important aspect of plant population dynamics directly linked with population persistence and colonisation of new areas.^31^ Forest trees can exhibit highly variable seed production, ranging from no seeds in some years to extremely high seed production in others. This variation is often synchronised within tree species in a local population and even among different tree species in the same forest.^11^ These patterns of intermittent, synchronous, population‐wide, mass production of seeds are termed ‘mast seeding’. This biological pattern was suggested to be linked to AM outbreaks in one study.^32^ Indeed, looking at the distribution of cases reported in several European countries from 2005 to 2015, a consistent, almost biennial pattern of increased incidence of AM cases was noted.^33^ Our data also showed annual variability in HGA concentration in sycamore seeds which might directly increase the risk of intoxication in some years compared with others. Masting is also intimately associated with flowering index and efficiency of pollination, which are additionally influenced by climatic variables such as temperature and rainfall.^11^, ^34^ Indeed, temperature during bud formation is closely associated with flowering duration and seed production,^8^, ^34^ but also it is paramount to guarantee viability during dormancy and to induce germination during spring. Rainfall, however, is negatively correlated with insect activity, hence decreasing pollination and subsequent seed crops.^11^ Although we did not look specifically at climatological data in this work, 2017 was generally warmer and wetter in early summer and autumn than in 2016; further, the 2018 summer was exceedingly warm and dry when compared with previous years.^35^ Interestingly, these observations correspond with favourable conditions^36^ for seed production and viability in sycamores and match with higher HGA concentration in sycamore seeds in 2017. In this work, we showed that trees growing close to one another had less variation in their seed HGA concentration than more distant trees, which supports local environmental influences on HGA production although genetic factors cannot be excluded as nearby trees might all be derived from closely related, or the same parent trees. More localised effects (such as sunlight exposure) within the same tree do not seem to play an important role in variability as we showed that overall, there was a low variability between bunches in the same tree and that HGA content was not influenced by the facing direction of seeds on a tree. A larger and more detailed meteorological study seems necessary to determine the impact on HGA concentration and annual sycamore seed production. Rates of pollination might be another important factor that might be associated with AM outbreaks given that unfertilised seeds and those with small or shrivelled embryos (flat seeds) had either low or undetectable HGA.

Plants are exposed to a range of environmental stresses, both abiotic and biotic, that act together to influence their biochemical constituents.^37^ Most notably, plant pathogens represent a ubiquitous biotic stress in the environment, which for *A. pseudoplatanus* include the tar‐spot leaf fungus (*Rhytisma acerinum*).^37^ This fungus is usually considered a harmless saprophyte that only causes damage when severe^38^: it is a common infection of otherwise healthy trees in most parts of the United Kingdom.^38^, ^39^ Despite the suggestion of an early postulated link between this fungal infection in *A. pseudoplatanus* leaves and AM cases,^25^ in our work, the presence of European Tar spot was not associated with HGA concentration in seeds of affected trees. Interestingly, several studies have shown that tar spot infection is more prevalent in countryside locations with good quality air: indeed, the presence of this fungus on maple leaves is frequently used as a bioindicator of lack of air pollution.^40^, ^41^, ^42^, ^43^ No association between seed HGA concentration and the location of the tree in urban areas was found, so it is unlikely that either *Rhytisma acerinum* infection and/or air pollution are associated with increased HGA production in sycamore seeds.

Soil composition is another abiotic factor that might influence seed development and tree growth. In this work, we did not find any association between soil type and HGA, but our comparison was based on soil maps rather than soil samples, so it is difficult to know whether a specific element was more abundant around high HGA producer trees. However, the annual variability noted within trees suggests that soil conditions are at least not a major contributory factor given that soil conditions would not change year to year in most cases.

Age partly determines the fertile life of trees and therefore the beginning of seed production. The age when a given tree starts producing seeds varies between species but it is around 25 years for *A. pseudoplatanus*,^8^, ^9^, ^44^ with higher seed production from 30 years onwards.^8^, ^12^ The presence of seed‐bearing trees will determine the availability of toxic material in pastures. This work showed a trend toward increasing HGA content of sycamore seeds as trees age, although our results did not reach statistical significance. Furthermore, our data showed evidence of increased *A. pseudoplatanus* colonisation in the United Kingdom since 1950. It is conceivable then that their increased numbers, and greater maturity over the past decades, account at least partially for the apparent rising incidence of AM in the United Kingdom. It would be interesting to determine whether the same increase in sycamore prevalence has occurred in other European countries over the same period.

In contrast to a previous study,^29^ trees within or near AM‐affected premises did not have a higher concentration of HGA than other trees. Methodological differences might account for this result. Westermann et al.^29^ analysed material directly encountered in affected pastures at the time of AM cases but the sampling in our work was performed retrospectively. Our follow‐up sampling over several years demonstrated that trees have a high annual variability of HGA concentration in their seeds, so the delay between AM cases and sampling might have affected our results. Interestingly, the tree with the highest concentration of HGA in its seeds in 2016 (503 ± 25.9 μg/g) was adjacent to a pasture with an AM case reported that same year.

The use of HGA by sycamores as a defence mechanism to avoid consumption by herbivores has previously been speculated.^45^, ^46^ We showed in this work that HGA increased in homogenised (but not whole) seed samples when exposed to increasing temperatures and moisture. It is possible then that production of HGA in sycamore seeds is triggered by browsing, or within crushed (perhaps trampled) seeds that lie on the ground. In cyanogenic plants, the toxin precursor and the enzymes that catalyse the reaction are located in different plant compartments to protect plant cells from cyanide toxicity: only when plant cells are damaged or stressed through crushing or chewing, the precursor glycoside comes in direct contact with the enzymes triggering the production of cyanide, which increases quickly.^47^, ^48^, ^49^ Our previous work also showed temporary increases in toxin production after sycamore seedling cutting.^46^ To our knowledge, the biochemical cellular pathways that result in HGA production by sycamores or related trees are not understood. However, most chemical defences are produced by plants as secondary metabolites: they tend to be recycled for nitrogen storage for plant growth as the risk of grazing declines.^47^ Hence, these compounds are often used in tissues that are vulnerable to grazing over short time spans: for example, in seedlings or seeds. Both are important for tree propagation and establishment and are more likely to be ingested inadvertently with surrounding grass; hence the possible requirement for higher HGA‐associated defences at this stage. Another finding that supports this HGA function is the presence of this compound in the seed fruit head exclusively, and the higher concentrations of HGA in tissues particularly important for propagation (seedlings and seeds vs. leaves^27^, ^29^).

We analysed a potential increase in HGA concentration as seeds ripen: AM outbreaks are typically associated with windy or stormy weather^16^, ^50^ and perhaps the presence of immature seeds that reach the ground prematurely could affect the toxic load of pastures in late summer or early autumn. However, we found no significant differences in seed HGA content between ripe or immature seeds. In contrast to the toxic potential of Red Maple leaves, in which the toxin, gallic acid increases in wilted leaves,^51^ the HGA content of sycamore leaves was lower in wilted autumn leaves than green spring leaves, whereas the HGA content of seeds remained stable. Consequently, wilted leaves appear to be a relatively minor source of HGA in autumn pastures, and the presence of partially ripened seeds does not increase the amount of toxic material in pasture according to our data.

The high dispersal and germination rate of sycamore seeds makes it difficult to control the presence of contaminated plant material in pastures for many horse owners. Indeed, sycamore seeds can be blown long distances (50–200 m),^52^ due to their winged shape. Further, they can fall into drinking water troughs or ponds used for livestock and, since HGA is soluble in water, contamination of troughs is a concern. This work showed that HGA contamination of water is possible when the water source contains seeds that have been frozen (but seemingly not with fresh seeds under the conditions studied); it is conceivable that freeze–thawing creates fissures in the seed surface that allows leaching of HGA, or ingress of water that increases HGA production, before leaching. However, the extent to which seed contamination of water causes toxicity will depend on the amount and duration of the seed contamination of a water source and the amount of water drunk. The results obtained in our experiment (with high seed contamination) revealed that the maximum tolerated dose suggested in equids by Valberg et al.^2^ and Boschnia et al.^1^ of 24.5 mg/horse, could be reached with the standard daily water intake of a horse. A 500 kg horse drinks on average around 30 L of water a day: ingesting contaminated water from freeze–thawed conditions (frost conditions) might lead to daily ingestion of 38 mg (high number of seeds experiment) or 8.7 mg (moderate number of seeds experiments) respectively. Hence, horses that drink from heavily contaminated water sources, particularly after frost might be at risk, and regular cleaning of water troughs is therefore recommended.

In summary, changes in HGA production in sycamore material are influenced by altered temperature and moisture levels primarily when the seed is damaged or disrupted, suggesting the possibility of its role as a plant defence mechanism in which browsing triggers increased production. Moreover, our data showed high annual variation in HGA production within the same tree suggesting that some climatological conditions favour HGA concentration; in contrast, none of the other environmental factors studied (fungal infections, air pollution or soil composition) significantly affected HGA concentration in sycamore seeds. Further study, with controlled plant growing conditions, might be necessary to dissect the precise climatic conditions that influence HGA production; likewise, longer‐term epidemiological examination of HGA seed data linked with climatic and location information, might help determine additional factors that influence mature tree seed HGA production. Further studies are required to decipher the biochemical pathways and mechanisms by which HGA is produced by sycamores and other trees.

## FUNDING INFORMATION

The Horse Trust and the Royal Veterinary College's Animal Care Trust.

## CONFLICT OF INTEREST STATEMENT

Prof. Piercy leads a commercial service that offers HGA and MCPA‐carnitine detection in serum and HGA in plant material for veterinarians and horse owners across Europe in collaboration with Dr. Carolyn Hyde. All income generated by the RVC's neuromuscular service supports the laboratory's research programmes. The remaining authors have no conflicting interests.

## AUTHOR CONTRIBUTIONS

**Sonia González‐Medina:** Investigation; methodology; formal analysis; data curation; writing – original draft. **Carolyn Hyde:** Writing – review and editing; resources. **Yu‐Mei Chang:** Writing – review and editing; software; formal analysis. **Richard J. Piercy:** Funding acquisition; writing – review and editing; project administration; supervision.

## DATA INTEGRITY STATEMENT

Sonia Gonzalez‐Medina had full access to all the data in the study and takes responsibility for the integrity of the data and the accuracy of data analysis.

## ETHICAL ANIMAL RESEARCH

Not applicable.

## ANTIMICROBIAL STEWARDSHIP POLICY

Not applicable.

## Supporting information


**Data S1.** Methods S1**—**Description of individual experiments.

## Data Availability

The data that support the findings of this study are openly available in 10.6084/m9.figshare.27992405.
