# Supplementary material for: Assessment of tree‐associated atypical myopathy risk factors in Acer pseudoplatanus (sycamore) seeds and leaves
Source: Equine Vet J. 2025 Jan 25;57(6):1656–65. doi: 10.1111/evj.14475 (PMC12508288; doi:10.1111/evj.14475)
Supplement: Supplementary file 1 — Data S1. Methods S1—Description of individual experiments. [file EVJ-57-1656-s001.pdf]

Equine Veterinary Journal BEVA

Methods S1

All plant material was ground to a fine powder under liquid nitrogen using an electronic grinder (Moulinex Multi Moulinette AT711) or a metallic pestle and mortar (individual samples) to create a homogenised sample.

Seed weight, maturity and seed parts experiment.

Approximately 150g of seeds were collected from 4 trees from at least 4 different samaras (bunch). Seeds were weighed and classified by the same observer as fully mature (dry), partially mature (green) or flat (non-existent or very small embryo) (Figure S1A). Hypoglycin A (HGA) concentration was individually measured in each of 20 seeds/tree within each category (mature, partially mature and flat).

Additionally, another 7 seeds/tree/category were sectioned as in Figure 1B with a scalpel, to separate the seed head (initially marked by pen) so that its maximum length could be measured. Full seeds and/or seed parts were ground to a fine powder under liquid nitrogen using a steel pestle and mortar and analysed for HGA content individually.

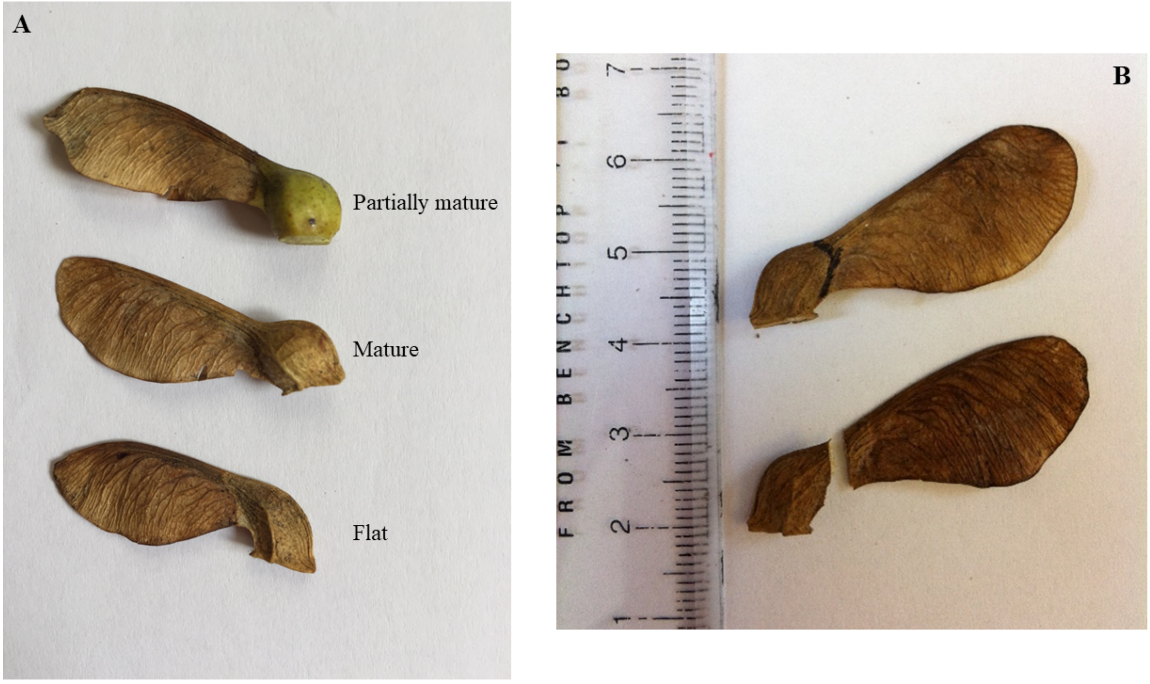

Fig S1: Individual seed experiments. A – different appearance of the samaras segregated for future analyses. B – sectioning of samara into the embryo and wing

Facing direction of seeds within the same tree experiment

Optimal *sample size* and adequate power to detect statistical significance was estimated from data presented by Valberg et al. (2013), using an HGA seed variation between 3µg/g and 160 µg/g. Based on a power of 80% and alpha of 0.05, 4 bunches of seeds from 3 trees would enable detection of a mean difference of 160µg/g.

Four trees were included in this experiment. Four seed bunches were collected from each tree from different branches (1 bunch/ branch) of each tree and the facing direction (North, South, East or West) was noted, based on the compass tool of an iPhone 6S. Five seeds per bunch (hence 20 seeds/tree) were weighed, processed and analysed for HGA concentration.

Data obtained were checked for normality through plotting of residuals and visual interpretation of a distribution histogram and subsequently log transformed. Data were analysed by Linear mixed effect model using SPSS 21 software. The statistical model included seed weight and location of samara within the tree as fixed effects while tree and samara within tree were allocated as random effects. Estimates of the covariance parameters (tree, bunch and residuals) were used to calculate the within and between tree HGA variation.

Association between HGA seed and leaf content and time of year

Approximately 150g of seeds were obtained from at least 4 different samaras in each collection. Collections were performed at 2 different times from the same 11 trees (last week of June and second week of October 2016). Approximately 150g of seeds were obtained from at least 4 different samaras in each collection. Logarithmic transformation of HGA content data was performed to achieve normality. A Student's paired t-test was used to assess variation of HGA over the maturity stages using GraphPad Prism 7.2 software.

Annual variation of HGA concentration within tree

Fourteen trees were included in this experiment. Approximately 150g of seeds were obtained from at least 4 different samaras in each collection. Collections were performed at the same time (15th October±5days) over 3 consecutive years (2016-2018). Data between years were compared by repeated measurement ANOVA. Statistical analyses were performed using GraphPad Prism 7.2 software.

Influence of local environment on seed HGA concentration

Thirty-two trees from 10 locations were included. Approximately 150g of seeds as previously described were collected between October and November (2016). All samples were run as technical duplicates. Data regarding the location of the tree (countryside/urban), presence of AM case/s reported within 130m (yes or no), presence of Tar spot fungus (*Rhytisma acerinum*) on leaves (yes or no) and trunk girth (a proxy for tree age) were noted. Trees were defined as growing within countryside if they were located a minimum of 100m from a village of fewer than 500 inhabitants and not adjacent to a major road. An urban location was assigned to all others. Tree girth measure was obtained at 1.5m height from the ground level, perpendicularly to the trunk axis with a measuring tape. Tree age was estimated by dividing the trunk girth measurement obtained by the average trunk growth rate of the tree species (in this case 2.5) as described by J White (1998).

Data concerning soil type in the sampling areas were obtained from the Cranfield soil and Agrifood institute website: www.landis.org.uk/soilscapes/index.cfm?panel=search. Soil types were grouped into 4 categories (base-rich, lime rich, slightly acid and loamy/clayey soil). All meteorological data were obtained from: www.metoffice.gov.uk/climate/uk/summaries.

Data were checked for normality through plotting of residuals and visual interpretation of a distribution histogram and log transformed when appropriate. They were then analysed by a General Linear Model using SPPS 21 software, using forward selection techniques, following linear regression screening to detect associations between logHGA content and each of the factors described above (fungal infection, AM cases, location, tree age). Only those factors that obtained $p \leq 0.6$ were included in the model. Additionally, the coefficient of variation of HGA seed content between trees within 10m from each other and between trees located at least 1km from each other was calculated.

Effect of temperature and moisture in HGA concentration in Sycamore seeds

Approximately 150g of seeds as previously described were collected from 6 trees from 5 different pastures the morning of the experiment. Three trees were collected the 2nd week of November 2016

and 3 trees the 2nd week of January 2017. All seeds from the same tree were pooled and ground to a fine powder under liquid nitrogen using an electronic grinder (Moulinex Multi Moulinette AT711) to create a homogenised sample. The resulting powder for each tree was then aliquoted into duplicated 1 g samples for 7 storage conditions for each tree. The conditions investigated were -80°C, -20°C; 4°C;20°C; 20°C, moist conditions (referred to as 'Moist') and 37°C, all for 48h. Moist conditions were created by adding 2ml of water. All conditions were run in technical duplicates and the mean result for each condition across each of the 6 trees calculated. Fresh (control) samples were processed directly. Statistical analysis of data obtained for the investigated conditions was conducted with SPSS software using a mixed effects model followed by Fisher's least significant difference (LSD) pairwise comparison, in which HGA concentration detected in each condition for each tree was compared to that of the fresh control sample.

After obtaining results for this experiment, a follow up experiment was designed to examine effect of temperature and moisture of the entire seeds (without homogenisation). Six trees were sampled the day of the experiment, collecting approximately 150g of seeds from 4 different bunches. Once in the laboratory, Falcon tubes were labelled with the temperature/moisture conditions used in the previous experiment and five seeds from each bunch (20 in total) were assigned to each of the same storage conditions for each tree and analysed similarly. A positive (homogenised) control sample was kept at 37ºC for 48 hours. Statistical analysis was conducted with SPSS software using a mixed effect model with a LSD pairwise comparison, in which HGA concentration detected in each condition for each tree was compared to the fresh control sample.

Water contamination with Sycamore seeds

The presence of seeds that fall into water troughs in pastures might represent a source of HGA intoxication for horses. This experiment was designed to replicate field conditions in a laboratory setting using Falcon tubes and deionised water. Each condition was examined in 3 technical replicates. Seeds from 3 different trees were added to water in 2 different numbers: 2 seeds/ in 10ml water (2s) and 4 seeds in 10ml water (4s) at laboratory room temperature (~20°C) for 4 days. Each of these conditions was evaluated when the seeds were either fresh (directly collected from trees) or after being frozen for 2 months, then thawed and added to the experimental tubes with water. When considering the large volume of most water troughs, both these experimental conditions represent very high contamination: for example, in a standard field water trough (~365litres), 2seeds condition will account for 73 000seeds/trough while 4seeds condition will account for 146 000 seeds.
